# Acute Encephalopathy Associated with Influenza A Infection in Adults

**DOI:** 10.3201/eid1601.090077

**Published:** 2010-01

**Authors:** Nelson Lee, Chun Kwok Wong, Paul K.S. Chan, Niklas Lindegardh, Nicholas J. White, Frederick G. Hayden, Edward H.C. Wong, Ka Shing Wong, Clive S. Cockram, Joseph J.Y. Sung, David S.C. Hui

**Affiliations:** The Chinese University of Hong Kong, Hong Kong Special Administrative Region, People’s Republic of China (N. Lee, C.K. Wong, P.K.S. Chan, E. Wong, K.S. Wong, C.S. Cockram, J.J.Y. Sung, D.S.C. Hui); Mahidol University, Bangkok, Thailand (N. Lindegardh, N.J. White); University of Oxford, Oxford, UK (N. Lindegardh, N.J. White); University of Virginia School of Medicine, Charlottesville, Virginia, USA (F.G. Hayden)

**Keywords:** Influenza A, influenza, infection, cytokine, encephalopathy, virus, adults, dispatch

## Abstract

We report acute encephalopathy associated with influenza A infection in 3 adults. We detected high cerebrospinal fluid (CSF) and plasma concentrations of CXCL8/IL-8 and CCL2/MCP-1 (CSF/plasma ratios >3), and interleukin-6, CXCL10/IP-10, but no evidence of viral neuroinvasion. Patients recovered without sequelae. Hyperactivated cytokine response may play a role in pathogenesis.

Influenza-associated acute encephalopathy has been described in children, and results in a high frequency of neurologic sequelae and death. Altered consciousness, disorientation, and seizures occur within a few days after the onset of fever and respiratory symptoms (*1**–**3*). In some patients, symptoms are transient but in others rapid progression to necrotizing encephalitis, deep coma, and death may occur (*1**–**3*). Cases in adults are infrequently reported and remain poorly characterized, although the more complex clinical scenarios in adults may have hindered case recognition (*1**,**4**–**6*). The pathogenesis is unclear, but a hyperactivated cytokine response, rather than viral invasion, is believed responsible in most childhood cases (*1**–**5*). We describe 3 cases of acute encephalopathy associated with influenza A infection in adults. The clinical, virologic, immunologic findings (cytokines in plasma and cerebrospinal fluid [CSF]), and CSF penetration of oseltamivir for these cases are reported.

## The Study

At Prince of Wales Hospital, Hong Kong (*7*), from January 2007 through August 2008, influenza infection was diagnosed for >460 hospitalized adult patients for whom acute febrile respiratory illnesses had been diagnosed. Nasopharyngeal aspiration and immunofluorescence assays (IFA) were used for rapid diagnosis of influenza A and B infection, confirmed by virus isolation. Thirteen (2.8%) patients had signs of confusion or altered consciousness, together with fever and respiratory symptoms (mean ± SD age 77.7 ± 8.8 years). We studied 3 patients from whom CSF was obtained for analysis, and who fulfilled the definition of influenza-associated acute encephalopathy (altered mental status >24 hours within 5 days of influenza onset and without alternative explanation) (*1**,**2**,**4**–**6*).

Nasopharyngeal aspirates were subjected to IFA, virus isolation, and subsequent subtyping (*7*). CSF specimens were subjected to virus isolation using MDCK cells, and reverse transcription–PCR to detect influenza virus RNA by using H1/H3 subtype-specific primers. Herpes simplex virus, herpes zoster virus, and enterovirus DNA/RNA was detected using PCRs (Technical Appendix).

CSF and plasma samples collected on the same day were analyzed simultaneously for the concentrations of 11 cytokines/chemokines by bead-based multiplex flow cytometry. Their assay methods and plasma reference ranges (established from >100 healthy persons) have been described (Technical Appendix) (*7*). In CSF, in patients without central nervous system (CNS) disease/infection, cytokines/chemokines are either undetectable (e.g., interleukin-6 [IL-6], CXCL8/IL-8, CXCL10/IP-10, CXCL9/MIG) or present at low levels (e.g., CCL2/MCP-1) (*8**–**10*).

Concentrations of oseltamivir phosphate (OP) and its biologically active metabolite oseltamivir carboxylate (OC) were measured in CSF and plasma taken simultaneously from 1 patient who received concurrent treatment, using tandem mass spectrometry (*11*). The assay methods have been described (Technical Appendix).

The clinical and virologic findings are summarized in Table 1. All case-patients were elderly (72–86 years of age), but none were known to have neuropsychiatric illness, dementia, or to be taking psychotropic medication. None had received updated influenza vaccination (*6*). Confusion and altered consciousness developed in patients 1 and 2 one to 2 days after the onset of fever and cough. These patients had no meningismus, focal neurologic deficit, hypotension, respiratory distress, or metabolic disturbances. Brain computed tomography (CT) scans showed no acute cerebral lesion. CSF analyses showed no bacterial or viral pathogen or pleocytosis. Oseltamivir was given to patient 2 only when influenza A was later confirmed by nasopharnygeal aspirate/IFA; patient 1 did not receive antiviral treatment. Both patients recovered in the next 2 days. Patient 3 had fever, severe chronic obstructive pulmonary disease exacerbation requiring noninvasive ventilatory support, complicated by acute coronary syndrome. He was given oseltamivir, 75 mg 2×/day, after influenza A infection was confirmed. Agitation and confusion developed in the patient on day 3–4 of illness (onset after the third dose of oseltamivir), despite resolution of the patient’s respiratory failure. These symptoms were followed by involuntary, tremulous movements involving all 4 limbs, while at rest and during movement. Brain CT scan was normal. Electroencephalogram showed generalized slowing. Oseltamivir was stopped after the ninth dose, but tremor persisted. CSF analyses showed no pathogen or pleocytosis. The patient’s symptoms resolved in the next 3–4 days without sequelae.

**Table 1:** Clinical and laboratory findings in 3 patients with acute encephalopathy associated with influenza infection, Prince of Wales Hospital, Hong Kong*

Clinical and laboratory findings	Patient 1	Patient 2	Patient 3
Age, y/sex	76/M	86/F	72/M
Concurrent illnesses	Ischemic heart disease	Diabetes mellitus, hypertension	COPD
Influenza vaccination within 6 mo	None	None	None
Symptoms on examination	Fever >38°C, cough, disorientation, incoherent speech, mental dullness	Fever >38°C, cough, delirious, impaired consciousness, did not follow verbal command	Fever >38°C, cough, disorientation, agitation, incoherent speech, involuntary 4-limb tremor
Focal neurologic sign or meningism	Absent	Absent	Absent
Chest radiograph, consolidation	Absent	Absent	Absent
Antiviral (oseltamvir)	None	Given	Given
Outcome (duration of encephalopathy)	Recovered (2–3 d)	Recovered (3–4 d)	Recovered (6–7 d)
Brain CT scan (noncontrast)	Normal	Old ischemic changes; known small, calcified meningioma	Normal
Virus isolated from NPA	Seasonal (H1N1) 2008	Subtype H3N2	Subtype H3N2
CSF testing results			
Opening pressure, cm H_2_O	11	9	14
Cell count (x 10^6^/L)	1	–	0
Glucose, mmol/L	4.2	7.4	3.7
Protein, g/L	0.46	0.47	0.16
Virus isolated	None	None	None
RT-PCR for H3 and H1 influenza virus	Negative	Negative	Negative
Bacterial culture	Negative	Negative	Negative
Others	HSV, HZV, and enterovirus PCR negative	HSV, HZV, and enterovirus PCR negative	HSV PCR negative

*COPD, chronic obstructive pulmonary disease; CT, computed tomographic scan; NPA, nasopharyngeal aspirate; CSF, cerebrospinal fluid; RT-PCR, reverse transcription–PCR; HSV, herpes simples virus; HZV, herpes zoster virus. In all cases, there was no hypoglycemia, and liver and renal function test results were normal. C-reactive protein level was elevated in all cases. For patient 3, an electroencephalogram was performed and showed generalized slowing of background consistent with moderate encephalopathic change (similar to that observed in septic encephalopathy) (*1*,*6*). Findings are consistent with previous reports on adult cases of influenza-associated encephalopathy: patients are all unvaccinated, pleocytosis and cerebral imaging abnormalities (even with magnetic resonance imaging) are usually absent, and symptoms are generally self-limiting (*1*,*6*). Most reports have mentioned influenza A as a cause of encephalopathy, and more commonly subtype H3N2 (*1*–*6*).

Despite apparently normal CSF findings, high concentrations of cytokines/chemokines were detected in the CSF and plasma specimens of all patients (Table 2). Plasma concentrations of IL-6, CXCL8/IL-8, CXCL10/IP-10, CCL2/MCP-1, and CXCL9/MIG were elevated at median values of 2.0, 2.8, 11.9, 3.7, and 2.1× the upper limits of their respective reference ranges (comparable to or higher than that observed in other hospitalized influenza patients) (Table 2) (*7*). Other cytokines were not elevated (*4**,**7*). In their CSF, IL-6, CXCL8/IL-8, CXCL10/IP-10, and CCL2/MCP-1 were consistently detected, and were elevated at median values of 2.6, 15.0, 3.4, and 20.0 × the upper limits of their respective plasma reference ranges. The CSF/plasma concentration ratios of CXCL8/IL-8 and CCL2/MCP-1 were >3 (median CSF/plasma ratio 5.4 and 8.0, respectively).

**Table 2:** Cytokine and chemokine concentrations in CSF and plasma samples from 3 patients with acute encephalopathy associated with influenza A infection, Prince of Wales Hospital, Hong Kong*

Cytokine or chemokine	Reference range, pg/mL	CSF/plasma cytokine concentration, pg/mL (ratio)		
		Patient 1	Patient 2	Patient 3
IL-6†	<3.1	8.0/6.3 (1.3)	11.6/35.1 (0.3)	2.2/5.9 (0.4)
CXCL8/IL-8‡	<5.0	84.0/15.5 (5.4)	74.8/13.8 (5.4)	21.9/6.3 (3.5)
CXCL10/IP-10†	202–1,480	15,374/102,019 (0.2)	5,101/17,594 (0.3)	1,371/1,550 (0.9)
CCL2/MCP-1‡	< 10-57	996/82 (12.1)	1,287/336 (3.8)	–
CXCL9/MIG	48–482	11,58/14,001 (0.1)	70/333 (0.2)	145/1,019 (0.1)
IFN-γ	<15.6	UD/14.4	4.7/10.1	0.4/2.0
IL-12p70	<7.8	1.5/UD	1.3/UD	UD/UD
TNF-α	<10.0	1.7/1.4	UD/1.2	UD/UD
IL-10	<7.8	2.5/2.2	UD/7.3	UD/1.7
IL-1β	<3.9	UD/UD	UD/3.7	UD/UD
CCL5/RANTES	4,382–18,783	4/2,507	14/1,609	1.3/814

*CSF, cerebrospinal fluid; –, test not done due to inadequate sample; UD, undetectable (i.e., below the detection limit of the cytokine/chemokine assay). Cytokines: Interleukin (IL)–1β, IL-6, IL-10, IL-12p70, tumor necrosis factor α (TNF-α). Chemokines: CXCL8/IL-8, monokine induced by interferon-γ (IFN- γ) (CXCL9/MIG), IFN-γ–inducible protein-10 (CXCL10/IP-10), monocyte chemoattractant protein–1 (CCL2/MCP-1), and regulated upon activation normal T cell–expressed and secreted (CCL5/RANTES). The plasma reference ranges are established from >100 healthy adults. The assay sensitivities of IL-1β, IL-6, IL-10, IL-12p70, TNF-α, IL8, MIG, IP-10, MCP-1, RANTES, and IFN-γ are 2.5, 3.3, 3.7, 1.9, 7.2, 0.2, 2.5, 2.8, 2.7, 1.0, and 7.1 pg/mL, respectively. Coefficients of variation are all <10%. In an earlier study involving 39 adult influenza patients hospitalized with cardio-respiratory complications (*8*), the median (interquartile range) plasma concentrations of IL-6, IL-8, IP-10, MCP-1, and MIG were 10.6 (4.2–18.4), 5.4 (2.5–8.7), 7,043.0 (4,025.1–1,2381.1), 76.5 (49.5-97.0), and 992.1 (499.1–1,992.3) pg/mL, respectively. In CSF, in subjects without neurologic disease/infection, these cytokines/chemokines are either undetectable or present at low levels (*9*–*11*). In a pediatrics influenza cohort, CSF cytokine levels were substantially higher in encephalopathy cases when compared to those with febrile seizure; CSF/plasma concentration was <1 (*9*). †CSF cytokine concentrations above plasma reference ranges. ‡CSF/plasma cytokine concentration ratio consistently >3 (3.5–12.1), in addition to CSF cytokine concentrations being above the plasma reference ranges. For IFN-γ, IL-12p70, TNF-α, IL-10, IL-1β and RANTES, because of their low/undetectable levels, the CSF/plasma ratios were not calculated. CSF specimens from patients 1 and 2 were collected at the peak of symptoms, and before antiviral treatment (if given); CSF from patient 3 was collected when persistent tremor developed 18 hours after the ninth dose of oseltamivir; the drug was stopped afterward.

Simultaneous CSF and plasma OC and OP concentrations were determined for patient 3, as symptoms progressed at 18 h after oseltamivir. The concentrations (mean ± SD) of OC in duplicate CSF and plasma samples were 18.3 ± 0.9 ng/mL and 143.8 ± 3.3 ng/mL, respectively; the CSF/plasma concentration ratio was 12%–13%. The OP plasma concentration was 1.05 ± 0.03 ng/mL; it was not detectable in the CSF.

## Conclusions

We report 3 adults with acute encephalopathy (altered consciousness, confusion) associated with influenza. High CSF and blood cytokine/chemokine (CXCL8/IL-8, CCL2/MCP-1, IL-6, CXCL10/IP-10) levels were detected. No evidence of direct viral neuroinvasion was found. All patients recovered rapidly without sequelae (*1**,**6*).

Our findings agree with studies of influenza-associated encephalopathy in children. Influenza virus is rarely detected in the CSF, and pleocytosis is often absent (*1**,**2**,**4**–**6*). High levels of cytokines (e.g., IL-6, soluble tumor necrosis factor receptor 1) can be consistently found in CSF/blood specimens, correlating with disease severity and outcomes (hyperactivated cytokine response is absent in febrile seizure associated with influenza) (*2**–**4**,**8*). We found a broader range of cytokines/chemokines being activated (*7*); for certain cytokines (CXCL8/IL-8, CCL2/MCP-1), the CSF concentrations were 3× those in plasma. IL-6, CXCL8/IL-8, CCL2/MCP-1 and CXCL10/IP-10 have been shown to play pathogenic roles in CNS viral infections, cerebral injury, and acute brain syndrome in susceptible patients (*7**,**9**,**12*). The high CSF/plasma ratios suggest that for some cytokines, activation within the CNS might have occurred along with respiratory-tract and systemic productions (cytokines are not detected in CSF normally; (Table 2) (*4**,**7**–**10**,**12*). Resident macrophages/monocytes, astrocytes, microglial and endothelial cells in the CNS are shown to release cytokines/chemokines when stimulated by viral/influenza infection; activation mechanisms without involving overt CNS invasion have been suggested (*1**,**4**,**9**,**12**–**14*). Cytokines may cause direct neurotoxic effects, cerebral metabolism changes, or breakdown of the blood-brain-barrier (endothelial injury) to produce symptoms (*1**–**4**,**8**,**12**–**14*). Whether early viral suppression by antivirals can lead to attenuation of these cytokine responses and better outcomes warrants further study (*7*).

We measured oseltamivir concentrations because of the concerns over its neuropsychiatric side-effects in children and adolescents. However, only the active metabolite (OC) was detected in the CSF of patient 3; the CSF/plasma concentration ratio was 12%–13% (18.3/143.8 ng/mL) at 18-hours postdose. This degree of CSF penetration is similar to that observed among healthy patients, with a Cmax CSF/plasma concentration ratio of 3.5% (at ≈8 hours), and a ratio of ≈10% at 18 hours (concentration-time profiles for plasma/CSF differ). Assuming a similar ratio, the CSF OP concentration would have fallen below the assay’s detection limit (0.25 ng/mL) by 18 hours (*11**,**15*). The low CSF drug-penetration, together with high cytokines in CSF and symptom progression despite drug withdrawal suggest that the manifestations of patient 3 may have been disease-related. Symptoms developed in patients 1 and 2 without antiviral exposure. Further investigations on the CNS effects of oseltamivir in the clinical setting are needed..

Our study is limited by the small patient number and the lack of feasibility in obtaining CSF for study/comparison in influenza patients without neurologic symptoms. Further studies on the clinical spectrum of influenza encephalopathy and encephalitis in adults (*1**,**6*) and their pathogenesis are indicated. In conclusion, acute encephalopathy may occur in adults with influenza. Exuberant cytokine/chemokine response may play an important role in its pathogenesis.

## Supplementary Material

Technical Appendix Acute Encephalopathy Associated with Influenza A Infection in Adults
