# Supplementary material for: Acute Encephalopathy Associated with Influenza A Infection in Adults
Source: Emerg Infect Dis. 2010 Jan;16(1):139–42. doi: 10.3201/eid1601.090077 (PMC2874350; doi:10.3201/eid1601.090077)
Supplement: Technical Appendix — Acute Encephalopathy Associated with Influenza A Infection in Adults [file 09-0077_Techapp-s1.pdf]

Acute Encephalopathy Associated with Influenza A Infection in Adults

Technical Appendix

Methods on Virus Detection and the Measurements of Cytokine and Oseltamivir Concentrations

Section I. Methods of Virus Detection

Influenza viral RNA was detected using H1/H3 subtype-specific primers (*1,2*) (H1: 5′-TGA GGG AGC AAT TGA GTT CA-3′ and 5′-TGC CTC AAA TAT TAT TGT GT-3′; H3: 5′-GCA ACT GTT ACC CTT ATG AT-3′ and 5′-TCA TTG TTT GGC ATA GTC AC-3′). After RNA extraction, a 10-µL aliquot of the extracted preparation was mixed with 15 µL of reverse transcription–PCR (RT-PCR) master mix, which containing 12.5 µL of 2× reaction mix, 0.5 µL of SuperScript III RT/ Platinum Taq Mix (Invitrogen, Carlsbad, CA, USA), 5 pmole of each forward and reverse primer, and adjusted to a volume of 15 µL with nuclease-free distilled water. For influenza A H1, the reaction condition was 30 min at 45°C for reverse transcription followed by 2 min at 94°C, and 30 cycles of 1 min at 94°C, 1 min at 52°C and 1 min at 68°C; and a final extension of 10 min at 68°C. The reaction condition for influenza A H3 was similar, except that reverse transcription was carried out at 50°C and the annealing temperature was 55°C. The PCR products were separated by gel electrophoresis and visualized by SYBR safe DNA stain (Invitrogen) under gel documentation system. The product size of influenza A H1 was 431 bp; the influenza A H3 product was 232 bp.

Enterovirus RNA was detected by one-step real-time RT-PCR (*3,4*). Amplification is specific for the 5′ untranslated region. Enterovirus RNA extracted from cell-culture fluid of clinical isolate confirmed by immunofluorescent was used as the source of positive control. RNA from the plasmid pAW109 was used as internal control for nucleic acid extraction as well as for the presence of inhibitors in the clinical samples. Primers and probes used were as follows: primer for enterovirus (EV), 5′-ACATGGTGTGAAGAGTCTATTGAGCT-3′, 5′-CCAAAGTAGTCGGTTCCGC-3′; primer for pAW, 5′-GCC TGG GTT CCC TGT TCC-3′, 5′-CGA CGT ACC CCT GAC ATG G-3′; probe for EV, 5′-(FAM)TCCGGCCCCTGAATGCGGCTAAT-3′ (TAMARA); and probe for pAW, 5′-(TET)CAGGCCAATGTCTCACCAAGC TCT-3′ (TAMARA).

Herpes Simplex Virus I (HSV1) was detected by real-time PCR (*5*). Amplification is specific for glycoprotein D. DNA extracted from cell-culture fluid confirmed positive for HSV-1 by immunofluorescent staining was used as the source of positive control. TaqMan Exogenous Internal Positive Control (EXO-IPC) DNA (Applied Biosystems, Foster City, CA, USA) was used as internal control for nucleic acid extraction as well as for the presence of inhibitors in the clinical samples. Primers used were 5′-CGGCCGTGTGACACTATCG-3′, 5′-CTCGTAAAATGGCCCCTCC-3′; TaqMan Probe: 5′FAM-CCATACCGACCACACCGACGAACC-TAMRA-3′.

Herpes simplex virus 2 (HSV2) was detected by real-time PCR (*5*). Amplification is specific for glycoprotein G. DNA extracted from cell-culture fluid confirmed positive for HSV2 by immunofluorescent staining was used as the source of positive control. TaqMan Exogenous Internal Positive Control (EXO-IPC) DNA (Applied Biosystems) was used as internal control for nucleic acid extraction as well as for the presence of inhibitors in the clinical samples. Primers

used were 5′-CGCTCTCGTAAATGCTTCCCT-3′ and 5′-TCTACCCACAACAGACCCACG-3′ (*4*)

Varicella zoster virus (VZV) was detected by real-time PCR (*5,6*). Amplification is specific for the diploid gene open reading frame 62 encoding for the major immediate transactivator. DNA extracted from cell-culture fluid of the VZV vaccine strain VarilRix (GlaxoSmithKline, Rixelsart, Belgium) or from clinical specimens confirmed positive for VZV by immunofluorescent staining was used as the source of positive control. TaqMan Exogenous Internal Positive Control (EXO-IPC) DNA (Applied Biosystems) was used as internal control for nucleic acid extraction as well as for the presence of inhibitors in the clinical samples. Primers used were 5′-TCTTGTCGAGGAGGCTTCTG-3′, 5′-TGTGTGTCCACCGGATGAT-3′; TaqMan Probe: 5′-TET-TCTCGACTGGCTGGGACTTGCG-TAMARA-3′.

Section 2. Measurement of Cytokine Concentration

Plasma concentrations of 10 cytokines and chemokines: interleukin(IL)-1β, IL-6, IL-10, IL-12p70, tumor-necrosis-factor (TNF)-α, CXCL8/IL-8, monokine induced by interferon-γ (IFN-γ) (CXCL9/MIG), IFN-γ-inducible protein-10 (CXCL10/IP-10), monocyte chemoattractant protein-1 (CCL2/MCP-1), and regulated upon activation normal T cell-expressed and secreted (CCL5/RANTES), were assayed using cytometric bead array (CBA) reagents (BD Pharmingen, San Diego, CA, USA) with 4-color FACSCalibur flow cytometer (BD Biosciences Corp, San Jose, CA, USA) (*2*). In CBA, different bead populations with distinct fluorescence intensities had been coated with capturing antibodies specific for different cytokines or chemokines. After incubation with 50 μL of plasma/cerebrospinal fluid (CSF), the cytokine/chemokine captured beads were mixed with phycoerythrin-conjugated detection antibodies to form sandwich complexes. Fluorescence flow cytometry of the beads provided simultaneous quantification of a panel of

cytokines and chemokines. IFN-γ was quantified by an ELISA (R & D Systems Inc., Minneapolis, MN, USA).

Section 3. Measurement of Oseltamivir Concentration

Oseltamivir concentration assays were performed by the Clinical Pharmacology Laboratory in the Faculty of Tropical Medicine, Bangkok, by using tandem mass spectrometry (*7*). Plasma and CSF samples were analyzed by using mixed mode (MPC-SD, 3M Empore Bracknell, UK) solid phase extraction and HILIC-LC-MS/MS. Stable isotope labeled (i.e., deuterated oseltamivir phosphate [OP] and oseltamivir carboxylate [OC]) internal standards were added to the samples before solid phase extraction. The drugs were quantified using an API 5000 triple quadrupole mass spectrometer (Applied Biosystems/MDS SCIEX, Foster City, CA, USA), with a TurboVTM ionisation source interface operated in the positive ion mode. Quantification was performed using selected reaction monitoring for the transitions m/z 313 – 225 and 316 – 228 for OP and deuterated OP, respectively, and 285 – 197 and 288 – 200 for OC and deuterated OC, respectively. The performance of the assay was demonstrated by analysis of 3 replicates of quality control samples at 3 levels. The coefficients of variation (CV%) during the analysis of OP were 4.1%,1.9% and 2.1% at 3 ng/mL, 20 ng/mL, and 150 ng/mL, respectively (limit of detection of OP, 0.25 ng/mL). The coefficients of variation (CV%) during the analysis of OC were 2.3%, 0.5%, and 3.1% at 30 ng/mL, 400 ng/mL, and 4,000 ng/mL, respectively.

References

1. Lee N, Chan PKS, Choi KW, Lui G, Wong B, Cockram CS, et al. Factors associated with early hospital discharge of adult influenza patients. Antivir Ther. 2007;12:501–8. PubMed

2. Lee N, Wong CK, Chan PKS, Lun SWM, Lui G, Wong B, et al. Hypercytokinemia and hyperactivation of phospho-p38 mitogen-activated protein kinase in severe human influenza A infections. Clin Infect Dis. 2007;45:723–31. PubMed DOI: 10.1086/520981

3. Dierssen U, Rehren F, Henke-Gendo C, Harste G, Heim A. Rapid routine detection of enterovirus RNA in cerebrospinal fluid by a one-step real-time RT-PCR assay. J Clin Virol. 2008;42:58–64. PubMed DOI: 10.1016/j.jcv.2007.11.016

4. Monpoeho S, Coste-Burel M, Costa-Mattioli M, Besse B, Chomel JJ, Billaudel S, et al. Application of a real-time polymerase chain reaction with internal positive control for detection and quantification of enterovirus in cerebrospinal fluid. Eur J Clin Microbiol Infect Dis. 2002;21:532–6. PubMed DOI: 10.1007/s10096-002-0766-5

5. Weidmann M, Meyer-König U, Hufert FT. Rapid detection of herpes simplex virus and varicella-zoster virus infections by real-time PCR. J Clin Microbiol. 2003;41:1565–8. PubMed DOI: 10.1128/JCM.41.4.1565-1568.2003

6. Wang K, Lau TY, Morales M, Mont EK, Straus SE. Laser-capture microdissection: refining estimates of the quantity and distribution of latent herpes simplex virus 1 and varicella-zoster virus DNA in human trigeminal ganglia at the single-cell level. J Virol. 2005;79:14079–87. PubMed DOI: 10.1128/JVI.79.22.14079-14087.2005

7. Lindegårdh N, Hanpithakpong W, Wattanagoon Y, Singhasivanon P, White NJ, Day NP. Development and validation of a liquid chromatographic-tandem mass spectrometric method for determination of oseltamivir and its metabolite oseltamivir carboxylate in plasma, saliva and urine. J Chromatogr B Analyt Technol Biomed Life Sci. 2007;859:74–83. PubMed DOI: 10.1016/j.jchromb.2007.09.018
